# Mitigating Spinal Cord Stimulator Lead Migration Complications in Minimally Invasive Spine Surgery: Technical Note

**DOI:** 10.7759/cureus.23343

**Published:** 2022-03-20

**Authors:** John P Walsh, Juan Jimenez

**Affiliations:** 1 Orthopaedic Surgery, Valley Hospital Medical Center, Las Vegas, USA; 2 Department of Neurosurgery, Riverside Medical Center, Kankakee, USA

**Keywords:** complications, hardware failure, paddle electrode, minimally invasive spine surgery (miss), spinal cord stimulation (scs)

## Abstract

Spinal Cord Stimulators (SCS) are a nonpharmacologic chronic pain management treatment modality that is well-validated and cost-effective within the surgeon’s armamentarium. The reported complication rates are between 5.3% to 40%, most commonly secondary to mechanical hardware failure. The most common mechanical complication is lead migration, which necessitates second surgery. The purpose of this technical note is to describe a minimally invasive spine surgery (MISS) implantation technique we believe to be more resilient to lead migration. We present a stepwise technique for SCS implantation with a maxillofacial screw and washer failsafe.

## Introduction

Spinal Cord Stimulators (SCS) are a nonpharmacologic chronic pain management treatment modality within the surgeon’s armamentarium that is well-validated and is cost-effective [1]. There are high levels of evidence to support SCS indications [2,3], but there is a paucity of literature rigorously characterizing risks and complications [4]. Descriptive studies report complication rates between 5.3% to 40% and are dominated by sporadic case reports of unique failure modes [2,5,6]. The incidence of technical failures and procedural complications in 68 studies of more than 2700 patients reported a lead migration incidence of 13.2%, well ahead of 9.1% for lead breakage as the second most common [7]. This parallels a review of the literature on the complications of SCS that identified hardware-related complications as more common than biological complications, of which lead migration was most common [8]. There exists an opportunity to ameliorate paddle lead migration through technical modifications [9]. 

SCS implantation techniques are broadly categorized into two categories: percutaneous and paddle lead implantation. Notably, the implantation of SCS is reported to have a low incidence of life-threatening complications in both percutaneous and surgical implantation [8,10,11]. Comparative studies have provided inconclusive evidence to support a superior modality [12], but surgical implantations of paddle leads are more common and the relevant approach for this technical note.

Minimally invasive spine surgery (MISS) has been found to reduce surgical-related morbidity by decreasing tissue damage, blood loss, and reduced pain relative to open approaches [13,14]. Leveraging the intrinsic benefits of MISS, surgical SCS implantation can more precisely place a mechanically robust construct. Further, an implant amenable to reversible extraction may reduce secondary surgical risks.

The purpose of this technical note is to present a stepwise technique for SCS implantation with a maxillofacial screw and washer failsafe to mitigate lead migration complications. We posit that prudent risk management should focus on minimal destruction of anatomy during paddle lead placement and have a robust but reversible fixation to reduce lead migration complications and accommodate the potential risk of reoperation. 

## Technical report

Eligibility for SCS implantation at our institution requires shared decision-making between the patient and the healthcare team. This collaborative effort begins with careful consideration of the procedure indications (Table 1) within the context of patient preferences and medical comorbidities by the senior surgeon (J.J), exhaustive conservative care, and medical management. Radiographs and magnetic resonance imaging may rule out residual pathoanatomy that may cause pain. The patient is required to undergo a successful trial procedure to isolate the most beneficial stimulation levels before the definitive implantation. This SCS implantation is a two-stage procedure, including a trial and implantation [15].

**Table 1:** Indications for Spinal Cord Stimulator (SCS)

Indications
Failed Back Surgery Syndrome
Complex Regional Pain Syndrome, Type I and II
Postherpetic Neuralgia
Trigeminal Neuralgia
HIV-Associated Pain
Pain After Amputation
Pain After Stroke
Multiple Sclerosis
Cancer-Related Pain
Diabetic Neuropathy
Spinal Cord Injury
Refractory Angina
Pelvic Pain

The patient is medically optimized and brought to the operative room. Following smooth induction of general anesthesia, the patient is placed in the prone position on a Trumpf table (Ditzingen, Germany). Fluoroscopy is brought into the field from the contralateral side, microscope ipsilateral, and microscope monitor at the foot of the bed. Before preparing and draping the thoracic and gluteal area, identify the operative level using fluoroscopy. The site is prepped and draped in a sterile fashion. A transverse incision is planned to provide access to the previously identified spinal levels: extend incision 1 or 2 vertebral body(s) below desired end location. Incision over T9 and T10 interspinous area will provide access to operate within T7-T8.

**Figure 1:**
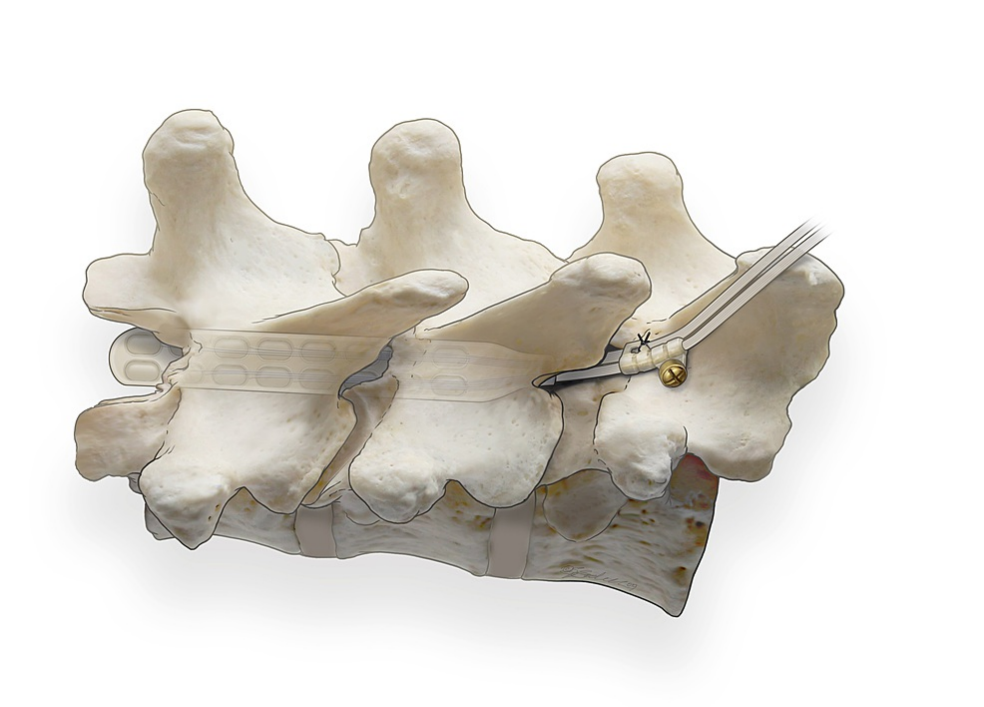
Screw and Washer Construct Fixation Screw and washer construct fixation at drill “groove,” near the anterior aspect of the lamina and immediately lateral to the spinous process. The groove will anchor the suture sleeve and stabilize paddle advancement. Focused drilling of the superior lamina obviates compromise of the facet complex. The construct will reduce torque on paddle leads and decrease the potential of migration and rotation.

Use rib counting on anteroposterior (AP) fluoroscopic views to identify levels used during the trial. After midline incision and before placing dilators, place two 2.0 silk sutures in the fascia without cutting or pulling off the needle. Insert sequential Medtronic (Fridley, MN) MetRx dilators. Drill a “groove” near the lamina's anterior aspect, immediately lateral to the spinous process (Figure 1). The groove will function as an anchor to the suture sleeve and stabilize paddle advancement. Focus drilling at the superior lamina to minimize the risk to the facet complex. The final construct will reduce torque on paddle leads and decrease migration and rotation potential. Next, drill along the lateral lamina to create a window for introducing the SCS paddle leads.

**Figure 2:**
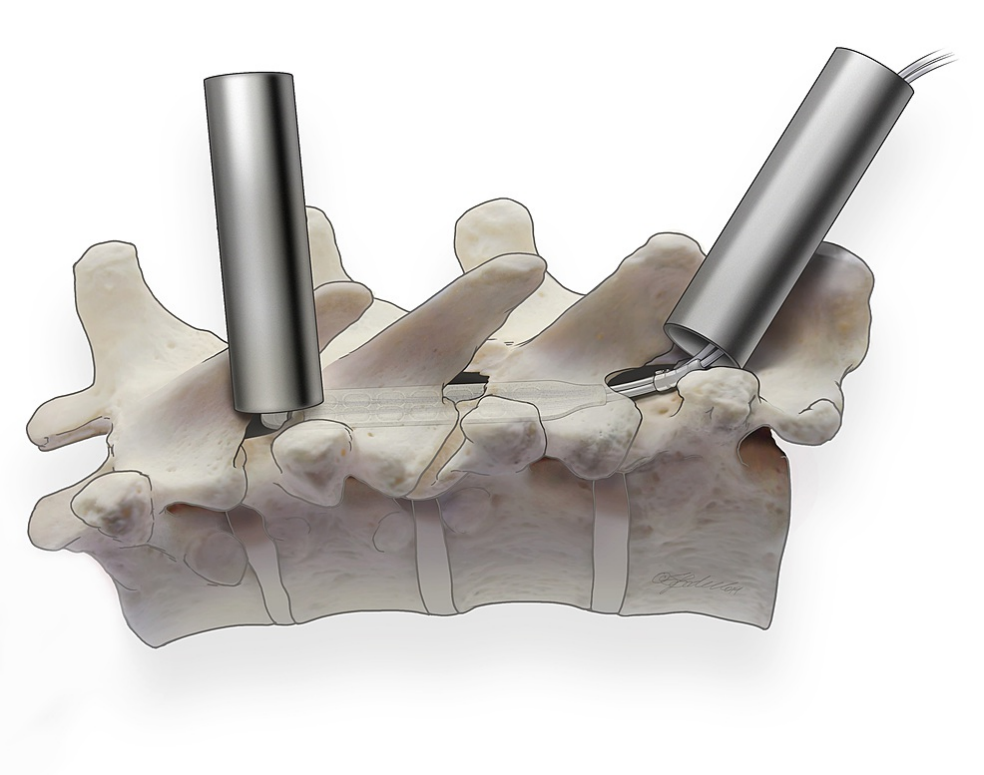
“Double-Barrell” Technique “Double-Barrell” approach to the management of recalcitrant leads. The second laminotomy provides improved visualization and physical access to paddle leads.

Obtain a fluoroscopic AP view to monitoring lead advancement, which will advance against mild resistance. Verify lead placement comparable to trial leads on fluoroscopic images, using both AP and lateral views. A scar-like formation may be present, likely secondary to trauma experienced during the trial. Use a brain spatula (Codman 59-1080) to remove obstructions in epidural space. Judiciously using Gelfoam to maintain adequate hemostasis during these maneuvers, hematoma formation has been reported [16]. Additional drilling and dissection may be required if non-invasive advancement attempts fail. Figure 2 illustrates the “double-barrel” approach to manage recalcitrant leads. The second laminotomy provides improved visualization and physical access to paddle leads. Secure 2cm anchoring device with 2.0 silk suture to wire contralateral to the side of the approach. Screws (1.5 mm x 5.0 mm or 2.0 mm x 6.0 mm) are placed with a self-drill through a 1.0 cm silicone suture sleeve until adequate bone fixation is achieved for securing the lamina. Irrigate the implant and remove the dilator tube. Remove the two left most wires and secure implant to click device. Secure anchors to interspinous ligament with 2-0 silk suture.

Prepare pocket for pulse generator in the gluteal area, according to the patient’s desires. Use the subcutaneous tunnel device to advance SCS wires from the mid-thoracic area toward the pulse generator. Close wound in a layered fashion, using 0 Vicryl for fascia, 2-0 for dermal layer, 4-0 monofilament for subcutaneous layer, and Dermabond for the skin. Take final AP and lateral fluoroscopic images to verify successful paddle lead implantation. 

## Discussion

We presented a stepwise technique for MISS SCS implantation with a maxillofacial screw and washer failsafe to mitigate lead migration complications. This approach is an adaptation made by the senior surgeon (J.J) in response to a complication pattern. The construct is durable and reversible, placed with a simple and reproducible technique. Establishing a gold-standard technique may standardize care to reduce variation and a more precise understanding of complications and mechanisms of failure. Since the etiology of lead migration remains unclear, we aim to minimize risks with reversible and MISS lead implantation. Pearls and potential pitfalls are noted in Table 2.

**Table 2:** Pearls and Pitfalls

Pearls	Potential Pitfalls
Increase the precision of paddle placement using the Roton micro tool to steer paddle lead from rostral laminotomy. To minimize torque on paddles and reduce the risk of paddle lead rotation or migration, create a "drill-groove" at the anterior aspect of the lamina- frees paddle lead course and anchor suture sleeve. “Double-Barrell” approach to overcome recalcitrant lead resistance. It provides visual and physical access to leads (Figure 2)	Inherent technical challenges due to tight and deep joint Pneumothorax. Minimize risk by using the tunnel technique from the thoracic incision toward the gluteal pocket Epidural scar obstruction. Restore midline lead placement with Codman 59-1080 brain spatula.

Henderson et al. determined technical factors that can substantially alter the reliability of SCS [17]. SCS optimization is dependent on adequate patient selection, implantation techniques, and stimulation parameters [2]. If reprogramming fails to restore baseline symptoms, surgical revision remains the only option to correct lead migration. Worse, surgical revision procedure outcomes reported in the literature are less favorable - nearly half of all patients that require revision will require multiple revision procedures. Thus, prevention remains a prudent strategy. 

Device manufacturers have devised their anchoring methods, but these continue to rely on suturing implanted leads to soft tissues or fascia. Tomycz et al. described a technique to incorporate bony anatomy using a titanium cranial plate for stability [18]. Our single screw and washer fixation is a distinct use of MISS, placing a slimmer and cheaper implant. Schwalb et al. reported that any increase in local trauma or scaring would exacerbate physiologic response and decrease SCS effectiveness [19]. Less augmentation of native anatomy is required and provides the option of reversal in a revision setting. 

Bone cement can stabilize the epidural exit point of the paddle electrode by reconstituting the removed portion of the lamina [20]. These authors demonstrated a 0% lead migration rate in their longitudinal follow-up. Pertinent drawbacks to consider are the increased operative time, cost, and risks associated with cement. Potential complications of this approach include compression of the thecal sac by cement extension into the spinal canal. This theoretical complication was not reported, nor any direct complications secondary to synthetic bone cement. If a revision procedure is required or implant complications develop, the removal of bone cement may place the spinal cord at risk. Screw and washer fixation provided an easily removable construct. 

Limitations of this publication include its lack of objective clinical data. We limited this description with technical details. Preliminary data portends a promising future, but a comparative investigation is required to investigate clinical significance in a future study. 

## Conclusions

This paper presented a stepwise technique for SCS implantation with a maxillofacial screw and washer failsafe. We believe this is a more durable and reversible implantation technique that is simple and reproducible. Evidence demonstrating the superiority of this technique is not reported.
